# Treatment after Progression on Fulvestrant among Metastatic Breast Cancer Patients in Clinical Practice: a Multicenter, Retrospective Study

**DOI:** 10.1038/s41598-018-37472-z

**Published:** 2019-02-08

**Authors:** Yizhao Xie, Yannan Zhao, Chengcheng Gong, Zhanhong Chen, Yinbin Zhang, Yanxia Zhao, Peng Yuan, Sainan Hu, Yi Li, Xichun Hu, Jian Zhang, Leiping Wang, Biyun Wang

**Affiliations:** 1 Department of Medical Oncology, Fudan University Shanghai Cancer Center, Department of Oncology, Shanghai Medical College, Fudan University, Shanghai, China; 2 Department of Breast Medical Oncology, Zhejiang Cancer Hospital, Zhejiang, China; 3 Department of Oncology, The Second Affiliated Hospital of Medical College, Xi’an Jiaotong University, Xi’an, Shanxi China; 4 Cancer Center, Union Hospital, Tongji Medical College, Huazhong University of Science and Technology, Wuhan, China; 5 National Cancer Center Tumor Hospital of the Chinese Academy of Medical Sciences, Beijing, China; 6 Department of Medical Oncology, Jiangsu Cancer Hospital, Jiangsu Institute of Cancer Research, Nanjing, Jiangsu China

## Abstract

Fulvestrant (Ful) is an effective and widely used agent for first- and second-line treatment of hormone receptor-positive, human epidermal growth factor receptor-2-negative (HR+/HER2−) metastatic breast cancer (MBC). However, there is no evidence of treatment after progression on Ful. Our study aimed to investigate the profile of daily practice regarding therapy after Ful. A consecutive series of 131 HR+, HER2- MBC patients who failed Ful 500 mg as first-line or second-line therapy from June 2014 to June 2017 in 6 institutions were included and analysed. Among 131 patients who failed Ful with similar baseline characteristics, 31 (23.7%) received endocrine therapy (ET), and 100 (76.3%) were treated with chemotherapy (CT). The most frequently applied CT regimen was capecitabine (32%), and the ET regimen was exemestane + everolimus (35.5%). Multivariate analysis showed that patients with bone-only metastasis were associated with lower CT use (OR = 7.97, 95% CI 1.51–41.84, P = 0.01). Among patients who received CT and ET as subsequent treatments, the median progression-free survival (PFS) was 7.5 months (95% CI 6.2–8.8) and 6.0 months (95% CI 4.1–7.9), respectively (p = 0.03). Among patients who were resistant to Ful (PFS < 6 months), the PFS on CT was significantly longer than that on ET (7.1 months vs 3.9 months, p = 0.024, HR = 0.5, 95% CI 0.26–0.97); however, among patients with a PFS ≥6 months on Ful, the efficacy of CT and ET was similar. Additionally, among patients with an older age, bone-only metastasis and ≥3 metastatic sites, no significant difference was observed between the CT and ET groups. Moreover, ET was much more tolerated than CT in terms of the incidence of grade 3/4 toxicities (9.6% vs 27%, P < 0.05). Median overall survival (OS) was not reached. Thus, our findings reveal the pattern of post-Ful treatment in current clinical practice and provide evidence on the efficacy, safety and choice of these treatments.

## Introduction

Breast cancer (BC) remains the most common cancer and cancer-related cause of death among women worldwide^1^. According to the latest data, 278.9 thousand women were newly diagnosed, and 66 thousand women died of breast cancer in China in 2014^2^. The morbidity and mortality of breast cancer has been increasing in recent years in China^2,3^, causing numerous social and economic burdens, especially for metastatic breast cancer (MBC).

Hormone receptor-positive (HR+) breast cancer accounts for nearly 70% of all BCs^4,5^. Among these patients, endocrine therapy (ET) is the first and foremost choice of treatment and can be sequentially given in several lines if patients have no evidence of symptomatic visceral metastasis, aggressive disease or endocrine therapy resistance^5^. Fulvestrant (Ful) is a selective oestrogen receptor downregulator. It works both by downregulating and by degrading the oestrogen receptor. In the phase III CONFIRM study, 500 mg Ful was proven to perform better than 250 mg Ful in estrogen receptor-positive (ER+) metastatic breast cancer (MBC) patients as a second line therapy^6^. In the FALCON study, 500 mg Ful showed better results than anastrozole for ER+ MBC patients as a first line therapy^7^. Based on this evidence, 500 mg Ful is the most effective single-agent endocrine therapy for patients who progress on initial endocrine therapy or who are endocrine therapy-naïve, and it is recommended and widely used in HR+ MBC patients in practice^5,8^. Although fulvestrant is often initially successful in the treatment of metastatic breast cancer, progression inevitably develops. What do we use after Ful? Should we switch to chemotherapy or continue endocrine therapy but with an agent with a different mechanism? Can we choose between these two therapies based on the PFS time on Ful?

However, few studies have answered these questions. Clinically, doctors’ choices are also controversial between another line of ET or chemotherapy (CT). This study aims to explore the choice of treatment after progression on fulvestrant and the efficacy and safety of post-Ful therapies in HR+/HER2− MBC patients in real world practice.

## Methods

### Patients

MBC patients who were treated with Ful between June 2014 and June 2017 were identified from databases from six institutions, including Fudan University Shanghai Cancer Center; The Second Affiliated Hospital of Medical College, Xi’an Jiaotong University; Union Hospital, Tongji Medical College, Huazhong University of Science and Technology; National Cancer Center Tumour Hospital of the Chinese Academy of Medical Sciences; and Jiangsu Cancer Hospital. Patients who had received subsequent treatment after progressing on Ful for at least one cycle and with a complete medical history were included in our analysis. All data were collected retrospectively from the medical records of individual institutions and managed by the Fudan University Shanghai Cancer Center. This study was approved by the Fudan University Shanghai Cancer Center Ethics Committee and Institutional Review Boards for clinical investigation. All of the methods were performed in accordance with the Declaration of Helsinki and the relevant guidelines. All of the patients signed written informed consent forms before inclusion in the study. This research is registered at clinicaltrials.gov (NCT 03541863).

### Study outcomes

Descriptive analysis included information on demographic characteristics, disease history, and treatment choices. Efficacy was assessed by progression-free survival (PFS) and overall survival (OS). PFS was defined as time from initiation of post-Ful treatment to disease progression. OS was defined as the time from initiation of post-Ful treatment to death from any cause until 20 April 2018. Tumour responses were confirmed according to Response Evaluation Criteria in Solid Tumors (RECIST) 1.1 criteria. Adverse events (AEs) were assessed according to the National Cancer Institute Common Terminology Criteria for Adverse Events (CTCAE) version 4.03.

### Statistics

Descriptive statistics were conducted to summarize patients’ characteristics as well as real-world practice of post-Ful therapy, which was categorized as CT or ET. Possible factors influencing the choice of treatment after Ful were evaluated by univariate and multivariate logistic regression. Prognostic factors regarding PFS and OS in different subgroups were investigated by a Cox regression model with a 95% confident interval (CI) in both univariate and multivariate models for all patients. The relation between PFS of Ful and treatment efficacy after Ful was explored using different cutoff points. Subgroup analysis was evaluated using the Cox regression model and expressed by forest plot, which compared the PFS of ET and CT in different subgroups, including age, metastatic status and line of Ful. Kaplan-Meier plots revealed the median PFS for each treatment, and a log-rank test was used to compare the PFS of two groups. A P value < 0.05 was considered statistically significant. Statistical analyses were managed using SPSS version 23.0.

## Results

### Patients and treatment

A total of 131 patients from six institutions with MBC who failed on first- or second-line treatment with Ful were evaluated for this analysis. Of these patients, 31 (23.7%) received ET after Ful, whereas 100 (76.3%) were treated with CT. Baseline characteristics of patients divided by different therapies are summarized in Table 1. The median age was 58 years for the ET (range 40–75) and CT (range 33–85) groups. For the ET and CT groups, most of the patients were postmenopausal (93.5% and 95%, respectively). Nearly half of the patients in the ET and CT groups had visceral metastasis (54.8% and 51%), received Ful as first line therapy (48.4% and 46%) and had a Ful PFS ≥6 months (54.8% and 45%). Previous endocrine use before Ful was similar between the two groups. Overall, there were no statistically significant differences in baseline characteristics between the two groups.

**Table 1:** Baseline characteristics of patients grouped by CT or ET.

Characteristics	Endocrine therapy N = 31 n (%)	Chemotherapy N = 100 n (%)	P values
Median Age	58	58	0.77
(range)	(40–75)	(33–85)	
**Menopausal status**			
Postmenopausal	29 (93.5)	95 (95)	0.754
Premenopausal	2 (6.5)	5 (5)	
**DFI**			
<2 years	4 (12.9)	21 (21)	0.593
≥2 years	25 (80.6)	74 (74)	
de novo stage IV breast cancer	2 (6.5)	5 (5)	
**ECOG score**			
0–1	30 (100)	97 (97)	0.631
≥2	0	3 (3)	
**Number of metastatic sites**			
1	9 (29)	33 (33)	0.94
2	12 (38.7)	32 (32)	
≥3	10 (32.2)	34 (34)	
**Metastatic sites**			
Visceral	17 (54.8)	51 (51)	0.709
Liver	10 (32.3)	18 (18)	0.13
Lung	12 (38.7)	41 (41)	0.82
Bone	19 (61.3)	63 (63)	0.864
**Line of Ful**			
1	15 (48.4)	46 (46)	0.723
2	16 (51.6)	54 (54)	
**Ful PFS**			
≥6 months	17 (54.8)	45 (45)	0.338
<6 months	14 (45.2)	55 (55)	
**Previous ET**			
Tamoxifen	15 (48.4)	50 (50)	0.875
NSAI	14 (45.2)	54 (54)	0.389
SAI	7 (22.6)	24 (24)	0.87

### Treatment choice and efficacy

The most frequently (≥10%) applied CT regimens after Ful were capecitabine, 32%; docetaxel-based, 19%; vinorelbine, 16%; and paclitaxel-based, 15%. Patients who underwent ET after Ful frequently received exemestane + everolimus (35.5%), exemestane (22.6%) or NSAI (12.9%).

Patients with bone metastasis alone were prescribed significantly less CT than ET (OR = 7.97, 95% CI 1.51–41.84, P = 0.01). No significant differences in choosing CT or ET were observed in univariate and multivariate models of patients with other characteristics, such as age, ECOG, DFI, visceral metastasis, metastasis sites, Ful as first-line or second-line therapy and PFS of Ful.

**Figure 1:**
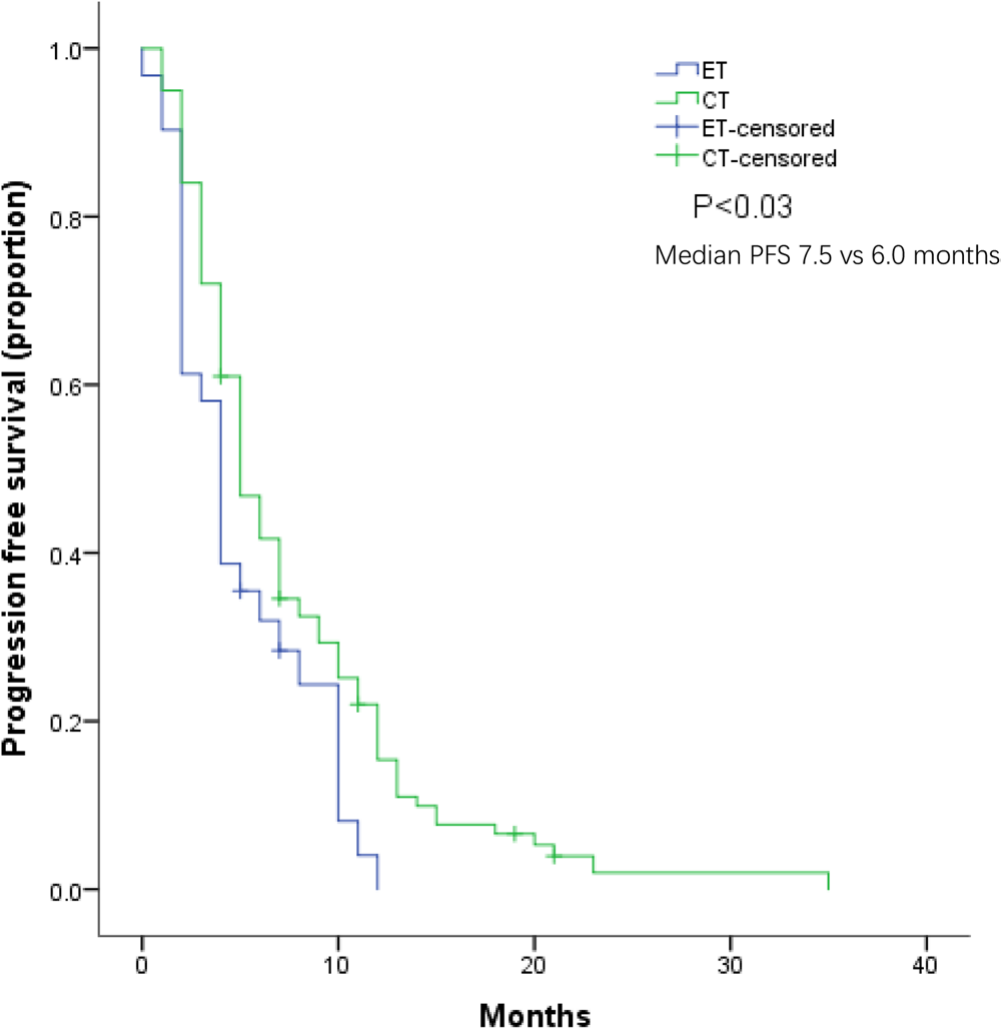
Kaplan–Meier curves for progression-free survival by treatment arm.

At a median follow-up of 14.5 months (range 5–20 months) of patients receiving CT and ET as subsequent treatment, the PFS was 7.5 months (95% CI 6.2–8.8) and 6.0 months (95% CI 4.1–7.9), respectively (p = 0.03) (Fig. 1). Among the ET group, patients receiving exemestane + everolimus had a median PFS of 6.0 months (95% CI 4.1–7.9), which was similar to that of CT (p = 0.29). The median OS was not reached at the time of analysis.

**Figure 2:**
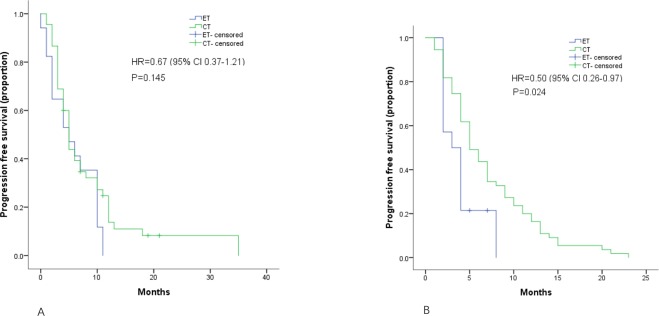
Kaplan–Meier curves for progression-free survival by treatment arm for patients with: A. Ful PFS ≥6 months B. Ful PFS <6 months.

Different durations of Ful were evaluated according to next line treatment efficacy, and a longer Ful PFS was finally defined at the cutoff point of 6 months. In patients who had a shorter Ful PFS (<6 months), the PFS of CT was significantly longer than that of ET (7.1 months vs 3.9 months, p = 0.024 HR = 0.5, 95% CI 0.26–0.97). However, in patients with a longer Ful PFS (≥6 months), the efficacy of CT and ET was similar (6.5 months vs 0.6.0 months, p = 0.145, HR = 0.67, 95% CI 0.37–1.21) (Fig. 2).

In the subgroup analysis, we found a significantly longer PFS in the CT group than in the ET group among patients aged ≤60 (7.8 vs 4.1 months, p = 0.002) and who received Ful as second-line therapy (8.2 vs 4.0 months, p = 0.004). Furthermore, no significant differences were observed for PFS between the groups among patients aged >60 (7.0 vs 6.0 months, p = 0.85), with bone-only disease (8.3 vs 7.0 months, p = 0.64), with ≥3 metastatic sites (6.3 vs 5.9 months, p = 0.72), and who received Ful as first-line therapy (6.9 vs 5.8 months, p = 0.52).

Baseline factors were evaluated to test the association with PFS, and we found no significant outcome differences.

### Safety

We evaluated the grade 3/4 adverse events (Table 2). Overall, grade 3/4 toxicities were more common in patients treated with CT than with ET (27% vs 9.6%, P < 0.05). Significantly more haematologic toxicities were observed in the CT group than in the ET group (23% vs 3.2%, P < 0.05). Palmar-plantar erythrodysesthesia syndrome, atrial fibrillation and increased alanine aminotransferase were seen in only the CT group (1% vs 0%). Pneumonitis was seen in both groups (1% vs 3.2%). Oral mucositis was observed in only the ET group (0% vs 3.2%), both of which were likely related to everolimus.

**Table 2:** Adverse events (grade 3/4).

AE (grade 3/4)	ET N = 31 n (%)	CT N = 100 n (%)
Leukopenia	0	17 (17)
Thrombocytopenia	0	3 (3)
Anorexia	0	2 (2)
Anemia	1 (3.2)	1 (1)
Palmar-plantar erythrodysesthesia syndrome	0	1 (1)
Atrial fibrillation	0	1 (1)
Alanine aminotransferase increased	0	1 (1)
Pneumonitis	1 (3.2)	1 (1)
Oral mucositis	1 (3.2)	0
All	3 (9.6)	27 (27)

## Discussion

Several studies have demonstrated the efficacy and safety of Ful in the real world. Naoko *et al*. reported a clinical benefit rate of 41.9% among 117 patients who received Ful in Japan^8^. Luca *et al*. showed good efficacy and tolerance of Ful in a prospective trial of 163 patients in Italy^9^.

However, a post-Ful study was absent. A review article indicated that there is no evidence after first- and second-line use of Ful^10^. The reason for this situation may be that 500 mg Ful was approved by the FDA in 2010 in the USA and by the cFDA in 2015 in China, Ful has a comparatively long disease control duration in first/second-line therapy, not many patients progress on fulvestrant, and there is limited experience we can refer to until now^6,7^. Some clinical trials included patients who failed on Ful. The BOLERO-2 study enrolled 119 (16%) patients previously treated with Ful, and exemestane + everolimus had a longer PFS than exemestane alone^11^.

This current study revealed the real-world practice of post-Ful treatment in ER+/HER2− MBC patients in China. We have determined that this is the first study focusing on therapy after progression on Ful, as well as on the daily practice of a post-Ful treatment pattern in the real world.

We found that more patients received CT than ET during post-Ful treatment. Doctors prescribed more ET for bone-only metastasis patients. We define the cutoff point of PFS for Ful as 6 months after exploring different cutoffs to support the use of next line ET. Despite the fact that CT had better efficacy than ET in certain subgroups, ET was as good as CT and had fewer grade 3–4 toxicities, which made the choice of ET reasonable for those patients. ET + everolimus can be a good option for ET.

The only factor that significantly influenced prescription choice was bone-only metastasis. Doctors tend to choose ET because of the lower tumour burden and a better prognosis for patients with bone-only metastasis. This trend was also seen in a study of first-line therapy^12^. Lobbezoo *et al*. reported that bone-only metastasis suggested an ET-first approach in their retrospective analysis^13^. Niikura *et al*. uncovered that ET was not inferior to CT in HR+/HER2− MBC patients with bone-only metastasis^14^. In our study, ET had a satisfactory result, which was similar in treatment efficacy to CT in patients with bone-only metastasis.

Capecitabine was the most common drug for the CT group. This fact shows that even when doctors decide to give a patient CT, they favour an oral and convenient agent. Martin M *et al*. reported that single-agent capecitabine had a median PFS of 3.7 months, a clinical benefit rate of 23% and good tolerance after pre-treating MBC patients in their phase III clinical trial^15^. The reason for choosing more CT than ET may be that no guidelines could be followed after Ful, there are limited targeted therapies available in China, patients may be less likely to benefit from further ET after first- or second-line ET and capecitabine is a convenient and tolerable CT agent.

As for the ET group, they predominantly received exemestane + everolimus. The BOLERO-2 study reported that patients previously treated with NSAI had a median PFS of 6.9 months after receiving exemestane + everolimus^11^. In our study, patients receiving exemestane + everolimus had a median PFS of 6.0 months, which was similar to the results of BOLERO-2. A noteworthy fact is that a CDK4/6 inhibitor is not available in China, and consequently, limited targeted therapies can be administered as post-Ful treatment. If available, we consider that CDK4/6 inhibitor-based regimens might be a favourable option for patients treated with fulvestrant.

Although more patients received CT than ET, their baseline characteristics were similar, making their survival results comparable. We found that CT was favourable to ET. Patients taking CT had a PFS of approximately 2.5 months longer than those taking ET (p < 0.03). However, no significant differences were observed in subgroups based on advanced age, bone-only metastasis, ≥3 metastatic sites, or Ful as first-line therapy, indicating that ET was equivalent to CT in these groups. Older patients may have more toxicities from CT and benefit less from CT than from ET. For patients with ≥3 metastatic sites, we think that their responses to ET and CT were poor due to comparatively extensive metastasis and greater tumour burden.

Since there is no definition of “Ful sensitivity”, we explored the relation between Ful PFS and efficacy of next-line ET, and we considered different cutoff times for Ful PFS. Finally, we found a PFS of 6 months or longer was the proper point to support the use of next-line ET. This definition can help doctors with the choice of post-Ful treatment according to Ful duration. Coincidentally, it is in accordance with the ABC consensus^16^. Furthermore, the PFS of patients receiving exemestane + everolimus was as good as that of those receiving CT, indicating that targeted therapy plus SAI can be a choice after Ful.

Prognostic analysis showed few differences between the two groups, probably because the baseline factors and tumour biological behaviour of these patients were similar.

As expected, the CT group had more 3/4 grade adverse events than the ET group, suggesting that patients who received CT may suffer more toxicities, need more visits or receive more symptomatic treatments in the hospital than those in the ET group. Meanwhile, we noticed 2 (6.4%) everolimus-related 3/4 grade AEs in the ET group that were also reported in BOLERO-2^11^, which reminds us of the potential toxicity of ET. A meta-analysis showed that 10.4% of everolimus recipients developed pneumonitis^17^.

Since the safety of ET exceeded that of CT, we recommend choosing ET in subgroups with similar treatment efficacy. In a study of first-line therapies, Bonotto *et al*. concluded that the median OS was nearly 3 years, regardless of CT or ET as a first-line treatment^18^.

In conclusion, this study explains the real-world pattern of post-Fulvestrant treatment, which was 76% CT and 24% ET. We define the cutoff point of sensitivity of Ful as 6 months. Despite the fact that CT was generally better than ET in certain subgroups, ET was as good as CT and had fewer grade 3–4 toxicities which made the choice of ET reasonable for those patients. Furthermore, ET+ targeted therapy can be a good option for ET. This study can help doctors with clinical decisions and help patients obtain maximum benefit.

In our study, all consecutive breast cancer patients who progressed on Ful and met our criteria were enrolled; however, due to the limited time since Ful has been on the market in China, as well as the relatively high price (approximately 1500$ per month), the sample size is limited, and the numbers of patients in the CT and ET groups are quite different, making it difficult to determine statistically significant differences. Moreover, the evidence level of a retrospective study is insufficient.

Randomized controlled trials (RCT) are warranted to provide more evidence on treatment after Fulvestrant in regions where CDK4/6 inhibitors are not available. Additionally, more effort is needed to find optimal sequences choose new endocrine therapy agents and enhance the life span of breast cancer patients.

## Conclusion

Our findings reveal the current pattern of post-Ful treatment in clinical practices in China. We observed that CT was more commonly selected by doctors than ET, probably because the lack of few evidence decreased confidence in ET following Ful. We explored the duration of Ful and defined a Ful PFS of over 6 months as a cutoff point giving support to the use of next-line ET. Moreover, in certain subgroups, ET was as good as CT and had fewer grade 3–4 toxicities, which made the choice of ET reasonable for those patients. For detailed agents, ET + targeted therapy can be a good option for ET after Ful. When CT was described, single-agent capecitabine was preferred.

## Data Availability

The datasets generated and analyzed during the current study are not publicly available due to hospital policy but are available from the corresponding author on reasonable request.
